# Biogeography shaped the metabolome of the genus *Espeletia*: a phytochemical perspective on an Andean adaptive radiation

**DOI:** 10.1038/s41598-017-09431-7

**Published:** 2017-08-18

**Authors:** Guillermo F. Padilla-González, Mauricio Diazgranados, Fernando B. Da Costa

**Affiliations:** 1 AsterBioChem Research Team, Laboratory of Pharmacognosy, School of Pharmaceutical Sciences of Ribeirão Preto, University of São Paulo, Ribeirão Preto, SP Brazil; 2 Natural Capital and Plant Health Department, Millennium Seed Bank, Royal Botanic Gardens Kew, Ardingly, West Sussex RH17 6TN UK

## Abstract

The páramo ecosystem has the highest rate of diversification across plant lineages on earth, of which the genus *Espeletia* (Asteraceae) is a prime example. The current distribution and molecular phylogeny of *Espeletia* suggest the influence of Andean geography and past climatic fluctuations on the diversification of this genus. However, molecular markers have failed to reveal subtle biogeographical trends in *Espeletia* diversification, and metabolomic evidence for allopatric segregation in plants has never been reported. Here, we present for the first time a metabolomics approach based on liquid chromatography-mass spectrometry for revealing subtle biogeographical trends in *Espeletia* diversification. We demonstrate that *Espeletia* lineages can be distinguished by means of different metabolic fingerprints correlated to the country of origin on a global scale and to the páramo massif on a regional scale. Distinctive patterns in the accumulation of secondary metabolites according to the main diversification centers of *Espeletia* are also identified and a comprehensive phytochemical characterization is reported. These findings demonstrate that a variation in the metabolic fingerprints of *Espeletia* lineages followed the biogeography of this genus, suggesting that our untargeted metabolomics approach can be potentially used as a model to understand the biogeographic history of additional plant groups in the páramo ecosystem.

## Introduction

After the final uplift of the Andean Cordillera, the northern region of the tropical Andes underwent a series of climatic fluctuations due to the successive glaciation and interglaciation events that occurred in the late Pliocene and early Pleistocene^1–3^. Consequently, a new ecosystem called the páramo emerged, combining the isothermal climatic conditions of the tropical region and the characteristic low temperature of the highlands^1–3^. Located between 3,000 and 4,800 m above sea level, the páramo extends mainly from northern Venezuela to Colombia, Ecuador and northern Peru^4, 5^. The páramos form a strongly fragmented ecosystem that functions biogeographically as an archipelago, with continental “islands” of open grassland vegetation separated by dense forests or deep Andean valleys^2, 5^, in which restricted species have little or no communication with species from distant páramos. This geographic isolation was especially defining for species with limited seed dispersal or long distance pollination and likely favored allopatric speciation processes^2, 3, 5^.

The Andean climate history was very dynamic. According to palynological records, several glaciation and interglaciation events during the Quaternary Ice Age forced the Andean vegetation belt into an almost continuous altitudinal fluctuation^1, 2^. The “upper forest line (at present at ca. 3200 m) shifted between 1800 m (glacials) and 3500 m (interglacials), corresponding to a variation in temperature between 5 and 15 °C at 2550 m altitude”^1^. This resulted in short periods of expansion of the páramo zone during the glaciations, allowing the colonization of new mountain systems and a continuous genetic flow among the páramo vegetation, followed by subsequent contractions of the páramo zone during interglaciations, resulting in the isolation of the vegetation^2^. This dynamic climate history is often considered a major driving factor in the remarkable diversification and rapid radiation of the páramo vegetation^6^.

The páramo is considered the fastest evolving biome on earth and the world’s most diverse high-altitude ecosystem^6–9^. Recent studies have demonstrated that 86% of the páramo angiosperm vegetation is endemic to this ecosystem and that many plant species are restricted to a single topographic area^10^. For instance, the Colombian páramos can be classified into five major biogeographic sectors, subdivided in 17 districts, 36 complexes, and 140 discrete units with distinctive patterns of angiosperm distributions^10–12^. This ecosystem holds the highest diversification rates of plant lineages, even compared to other rapidly evolving ecosystems such as the Hawaiian Archipelago and the Brazilian Cerrado^6^. Among the páramo plant lineages with the fastest diversification rates, the subtribe Espeletiinae (Asteraceae) represents a prime example^6^. Espeletiinae constitutes a group of 144 taxa classified into eight genera: *Carramboa* Cuatrec., *Coespeletia* Cuatrec., *Espeletia* Mutis ex Bonpl., *Espeletiopsis* Cuatrec., *Libanothamnus* Ernst, *Paramiflos* Cuatrec., *Ruilopezia* Cuatrec., and *Tamania* Cuatrec^13, 14^. With 72 species currently described, *Espeletia* is the most diverse genus within Espeletiinae^15^ and its abundance in the páramo makes it a representative taxonomic group of páramo plant life and an outstanding example of adaptive success^5, 13, 14^. Therefore, *Espeletia* is usually considered an ideal model for studying rapid adaptive radiations and evolutionary plant processes^3, 5, 16^.

**Figure 1:**
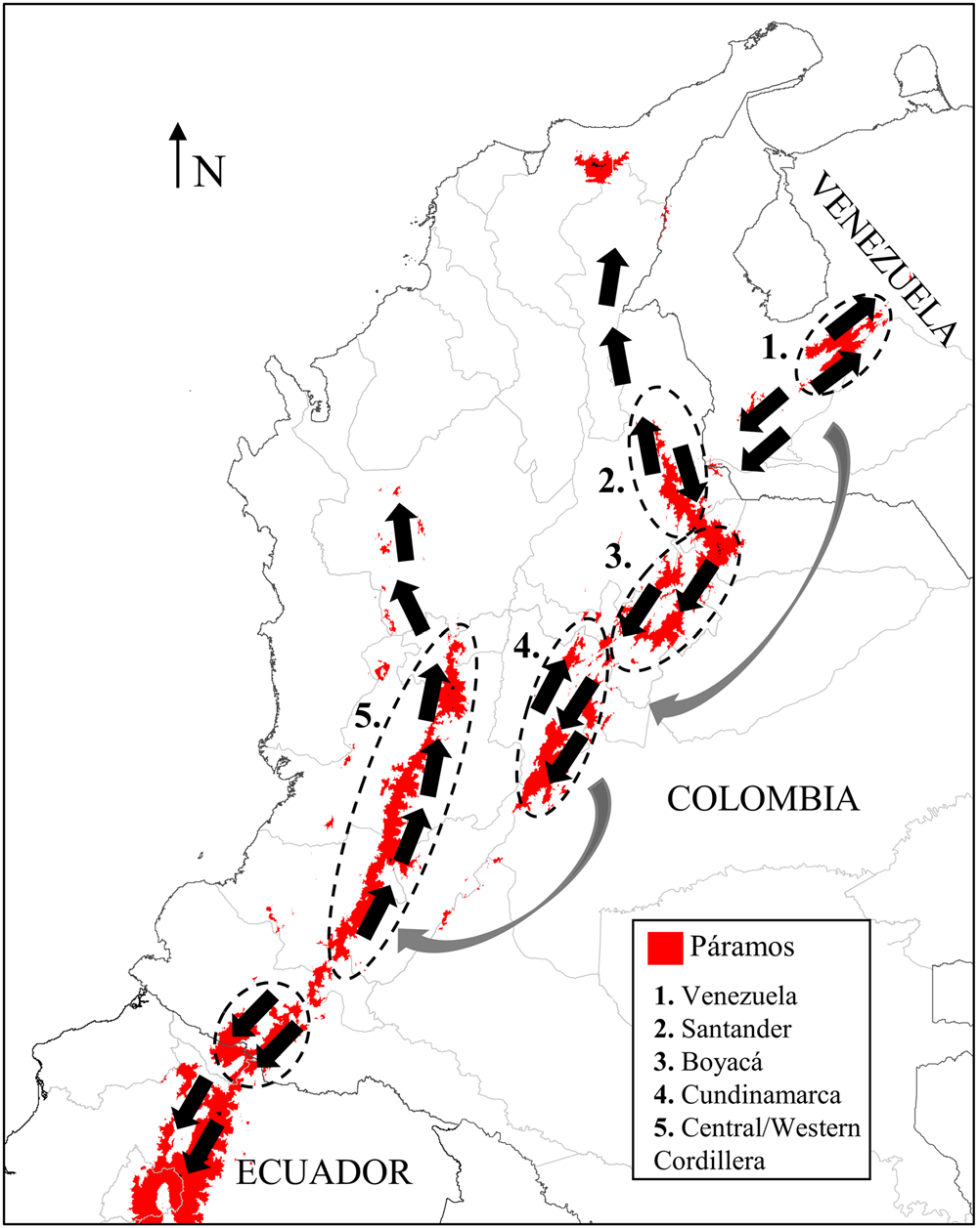
Migration routes of the genus *Espeletia* proposed by Cuatrecasas (2013) from its center of origin in Venezuela. Dashed circles correspond to the main centers of biogeographic diversification. Map created using DIVA-GIS software, version 7.5.0.0 available at http://www.diva-gis.org.

One of the first studies on *Espeletia* reported that different geographic areas in the Colombian Andes differ in species composition, concluding that the taxonomy of this genus was strongly influenced by the geography^17^. Recently, an elaborate hypothesis regarding the origin and migration routes of *Espeletia* along the northern Andes was proposed based only on their distribution patterns and ecological data^5^. The original stock of *Espeletia* diversified when the first population of the genus started expanding in two directions from the western part of the Cordillera de Mérida in Venezuela. One population branch moved along the Venezuelan Andes, whereas the other moved towards the west and southwest along and across the Colombian Andes, and into northern Ecuador^5^ (Fig. 1). This hypothesis was partially supported by molecular markers^18^, highlighting the influence of geography and past climate history on the diversification of the group^5^. Although some morphological characteristics of *Espeletia*, such as the spiral leaf phyllotaxis, the size and type of inflorescence, the shape of the leaves and the type of indument, are suggested to have been shaped by biogeography^5^. However, there is little evidence supporting the influence of biogeography on these or other phenotypic characteristics.

All existing studies investigating the geographical isolation of the páramo vegetation and their biogeographical trends are based exclusively on genetic and morphological information^5, 19^. For example, a recent study based on amplified fragment-length polymorphisms (AFLPs) and plastid DNA markers revealed two major biogeographical groups of species in the Andean-endemic genus *Loricaria* (Asteraceae), concluding that a complex interplay of biogeography, past climate history and local ecological conditions are the driving forces of diversification in plant species of this genus^19^. However, molecular markers have failed to reveal subtle biogeographical trends in the diversification of most of the páramo vegetation, and in the genus *Espeletia*, only two well-supported clades related to the species’ country of origin (Colombia or Venezuela) were recovered when performing concatenated analyses including AFLPs, ITS, ETS and rpl16 markers^18^. The low variation of conventional DNA markers, the incomplete lineage sorting of alleles and the frequent hybridization of *Espeletia* species add additional constraints to the use of molecular markers for identifying subtle biogeographical trends and offer limited possibilities for addressing recent evolutionary events.

Secondary metabolites (SM) contribute to the adaptive success of a plant species by serving as a chemical interface between the plant and its environment^20^. The comparison of the chemical fingerprints of plants by means of multivariate statistical tools may provide valuable information regarding the possible relationship between the biogeographical history and the phenotype of the páramo vegetation. In this context, metabolomics constitutes an interesting approach for studying the whole set of metabolites within an organism and has been previously used to trace the influence of different abiotic environmental factors on a plant’s metabolite profile^20^ and to infer chemotaxonomic relationships between different plants or microorganisms^21, 22^. Despite the fact that evidence for biogeographic isolation of extremophilic bacteria has been obtained using metabolomics approaches^23^, to the best of our knowledge, phenotypic evidence for allopatric segregation in plants at a metabolomics level of comparison has never been reported.

Here, we present for the first time a metabolomics approach based on ultrahigh-performance liquid chromatography coupled to UV detection and high-resolution mass spectrometry (UHPLC-UV-HRMS) to reveal subtle biogeographical trends in plant diversification. We aimed to (1) employ a UHPLC-UV-HRMS-based untargeted metabolomics approach to study the metabolic fingerprint of the model genus *Espeletia* and correlate the metabolic data with the main diversification centers of this genus; (2) identify patterns in the accumulation of secondary metabolites according to the main diversification centers of *Espeletia*; and (3) perform a comprehensive phytochemical characterization of this genus.

## Results

### UHPLC-UV-HRMS-based metabolic fingerprinting and biogeographic correlation

UHPLC-UV-HRMS analysis of 120 plant extracts from the genus *Espeletia* resulted in 3,661 and 2,554 mass features in the positive and negative ionization modes, respectively. The negative mode dataset was chosen for further analyses and an average of 212 mass features per sample were initially detected in this mode (Table S2). After removal of isotopes, an average of 148 mass values per sample remained (Table S2). Prior to statistical analysis, the peaks detected in the blank were subtracted from each plant sample and 28 missing values were manually removed from the original matrix. Two types of multivariate analyses were performed with all detected mass features: unsupervised methods by hierarchical clustering analysis with bootstrap resampling (HCAbp) and non-metric multidimensional scaling (NMDS), and a supervised method using orthogonal projection to latent structures discriminant analysis (OPLS-DA).

**Figure 2:**
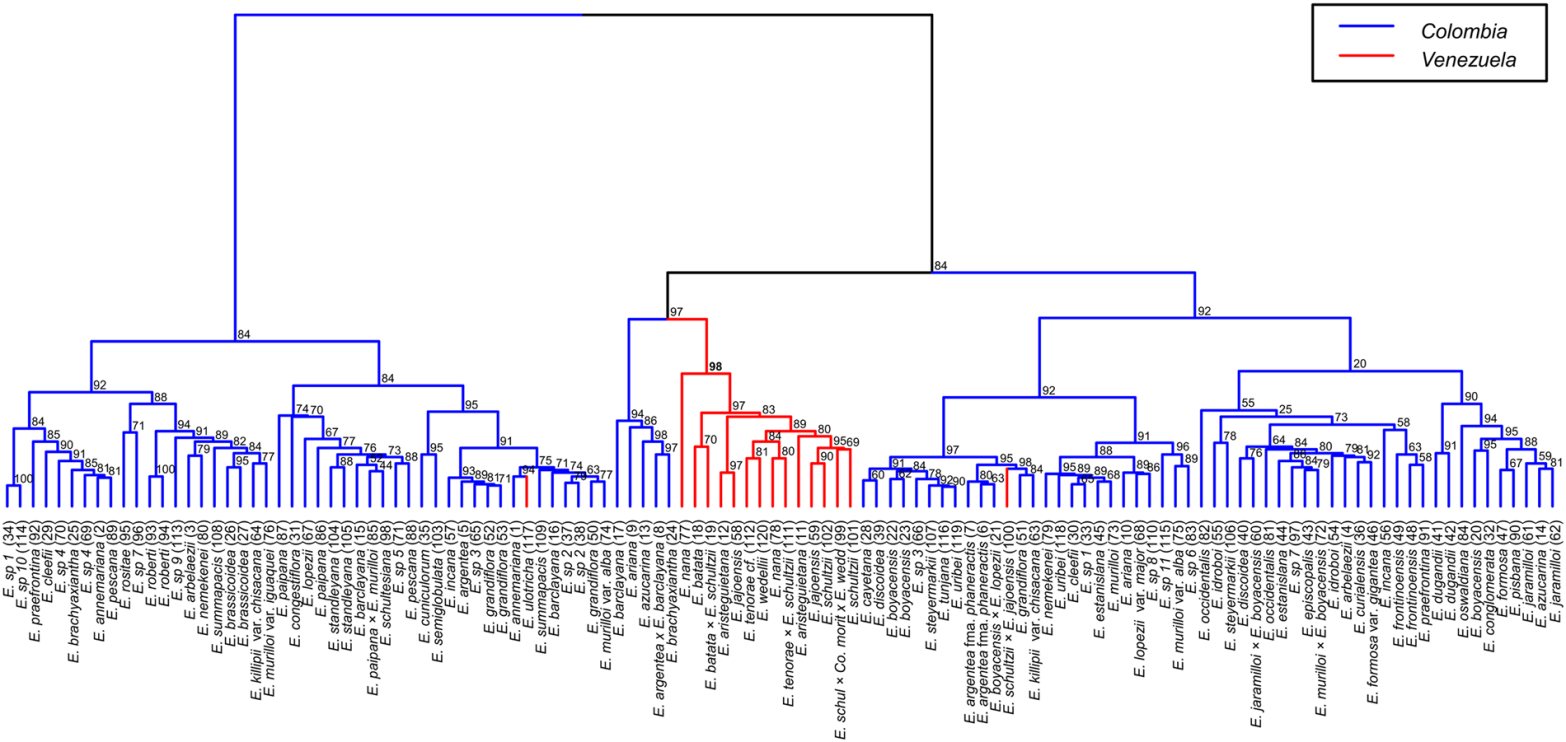
HCAbp based on metabolic fingerprinting in negative ionization mode of 120 plant samples of the genus *Espeletia* analyzed by UHPLC-UV-HRMS.

HCAbp of the UHPLC-UV-HRMS negative mode dataset grouped plant extracts by chemical fingerprint similarity (Fig. 2). This analysis revealed a species-grouping tendency according to the country of origin (Colombia or Venezuela; Fig. 2). With a high-unbiased *p* value of 98%, most of the species from Venezuela were grouped in a single cluster (Fig. 1), while Colombian species were grouped in different clusters.

**Figure 3:**
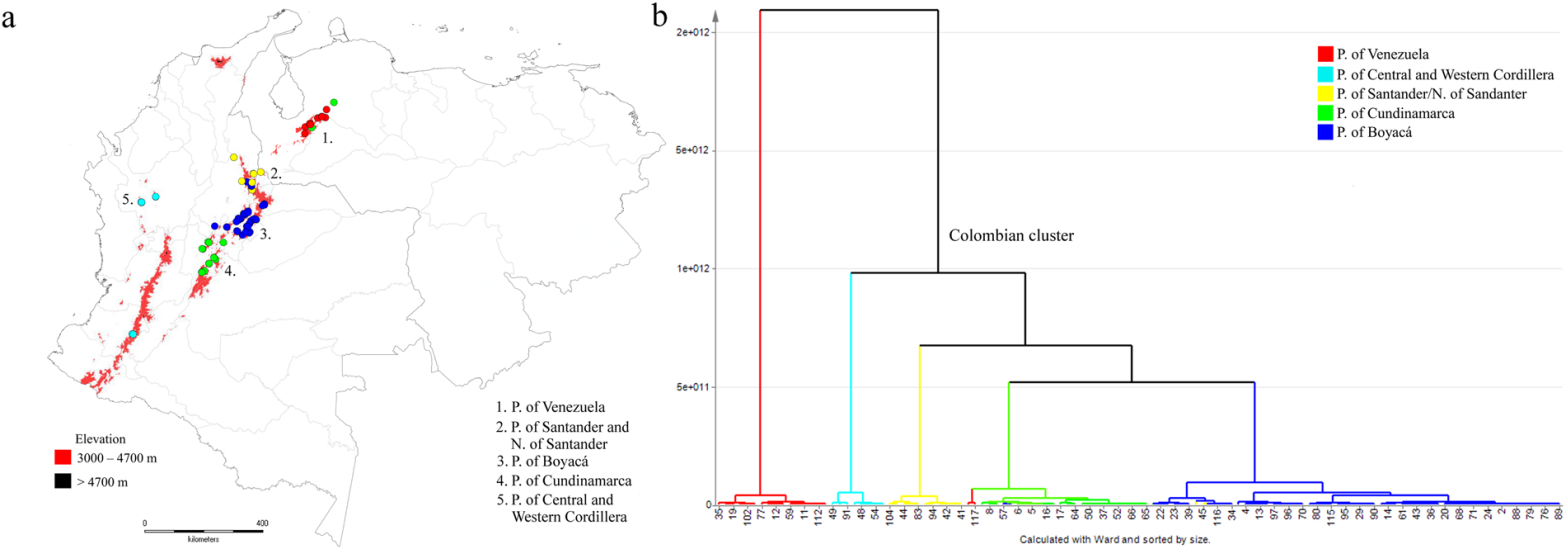
Clustering of *Espeletia* species based on metabolic fingerprinting in negative ionization mode of 120 plant samples analyzed by UHPLC-UV-HRMS. (**a**) Map of Colombia and Venezuela with species (represented as dots) colored according to their OPLS-DA groupings and (**b**) OPLS-DA dendrogram showing the clustering of species according to their páramo massifs of origin. Map created using DIVA-GIS software, version 7.5.0.0 available at http://www.diva-gis.org.

The OPLS-DA model, with five major diversification centers of *Espeletia* ^5^ as the Y variable (R^2^ = 0.82, Q^2^ = 0.52, CV-ANOVA = 1.13E-28, number of components = 4 + 6 + 0, Fig. 3b), revealed important differences in the metabolic fingerprints of the species according to their páramo localities. Four of them corresponded to Colombian massifs (Boyacá, Cundinamarca, Santander/Norte de Santander and Central and Western Cordillera) and one resembled a Venezuelan massif (Venezuela). Interestingly, the chemical fingerprint-based clustering reflects the geographic proximity of these páramo massifs (Fig. 3a). Species from the páramos of Boyacá, Cundinamarca, Santander and Norte de Santander were clustered together (Fig. 3a). Nevertheless, in some cases, species located in neighboring geographic areas were grouped into adjacent clusters, suggesting that they present similar metabolic fingerprints. For example, three species from Santander, *E. brassicoidea* (27), *E. estanislana* (45) and *E. steyermarkii* (107), and one from Cundinamarca, *E. uribei* (118) were grouped in the Boyacá cluster (Fig. 3a).

**Figure 4:**
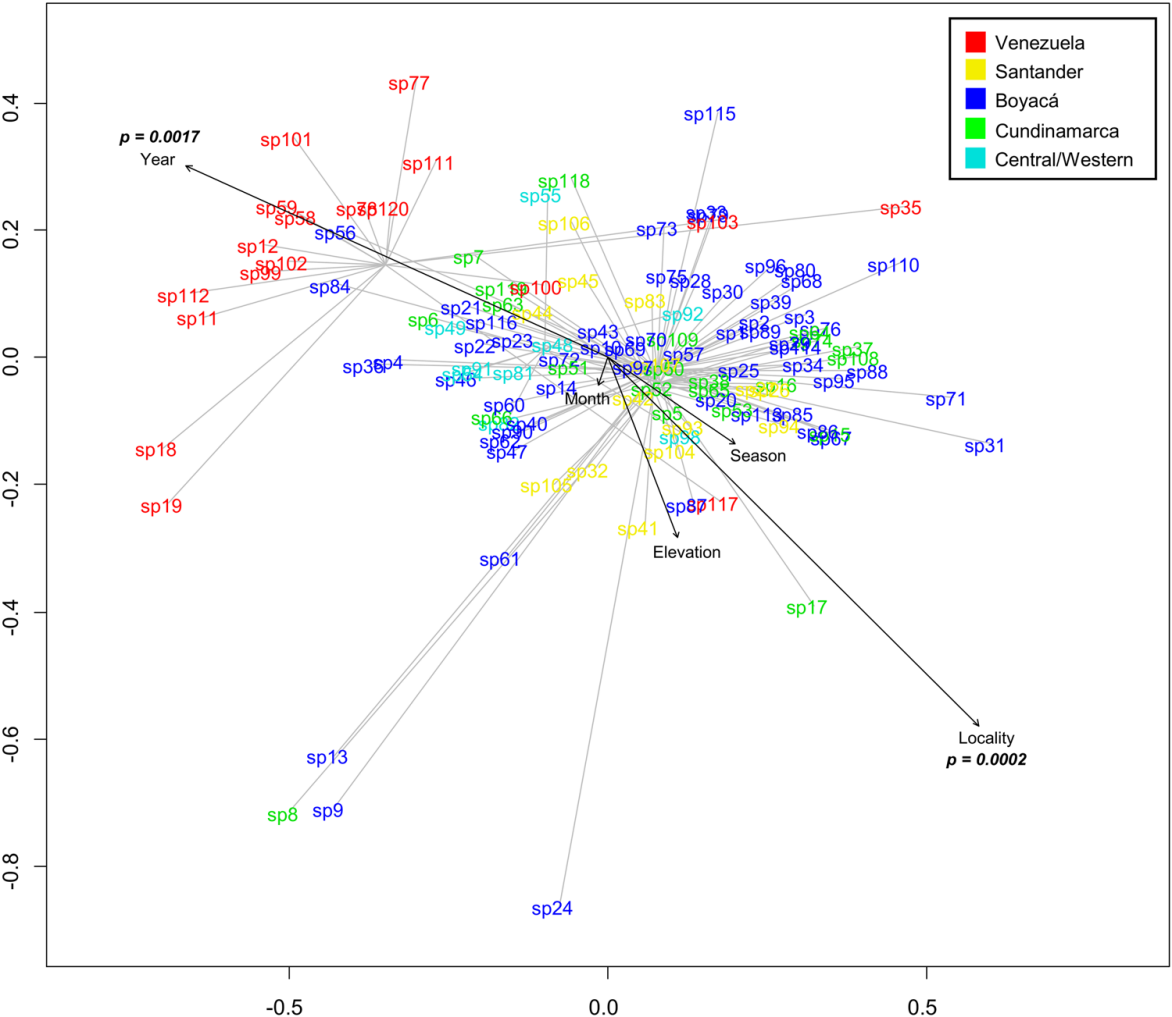
NMDS based on metabolic fingerprinting in negative ionization mode of 120 plant samples of the genus *Espeletia* analyzed by UHPLC-UV-HRMS, showing the correlation of environmental factors with the clustering of species by geographical origin.

To assess the significance of the locality, season, elevation and date of collection on the species’ metabolic fingerprints (Table S2), the data matrix was subjected to NMDS using the Bray-Curtis dissimilarity index (Fig. 4). This method revealed that among the studied factors, only the locality and year of collection were statistically significant (locality, *p = *0.0002; year, *p* = 0.0021).

### Accumulation patterns of SM according to *Espeletia* diversification centers

**Figure 5:**
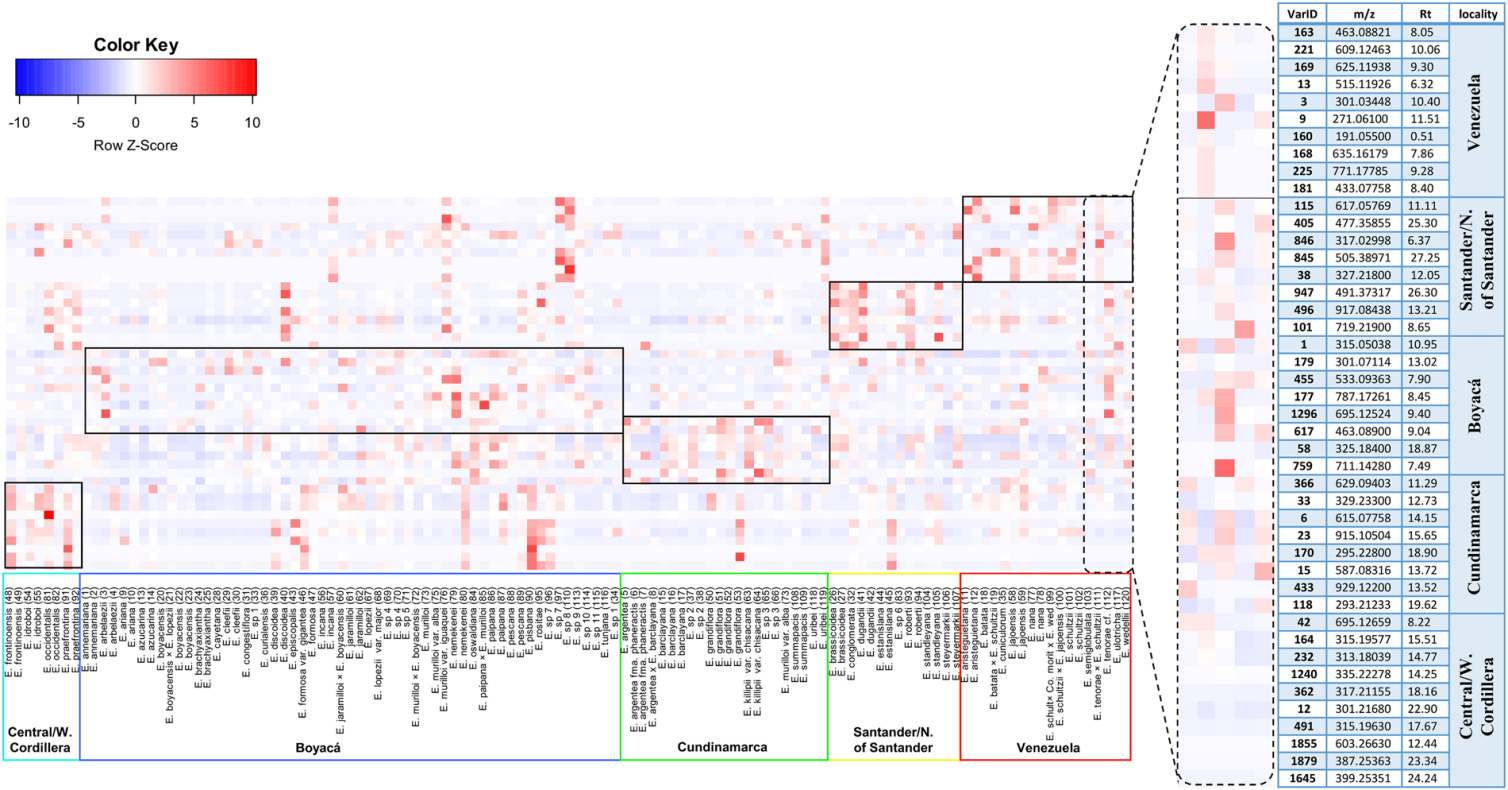
Heatmap based on metabolic fingerprinting in negative ionization mode of 120 plant samples of the genus *Espeletia* analyzed by UHPLC-UV-HRMS, showing the differential accumulation of the top ranked OPLS-DA mass features (right box) according to each geographic locality (for compound identities see Table 1).

The OPLS-DA-based model (Fig. 3b) revealed a differential accumulation of metabolites by species from the same páramo massif. The heatmap based on the OPLS-DA top-ranked mass features by geographic locality (Fig. 5) showed that species belonging to each páramo massif differed in their metabolic expression, with some species or groups of species (e.g., those from Boyacá) showing a more heterogeneous metabolic expression than other groups. Accumulation patterns of SM was performed only with the OPLS-DA top-ranked mass features to validate the biomarkers selected by the algorithm and graphically representing the species that produce the discriminating metabolites at higher concentrations (Fig. 5 and Table 1), therefore serving as basis for further studies. Species belonging to the páramos of Santander and Norte de Santander (Fig. 3a) were the only group that contained putative triterpenes as the main discriminatory substances correlated with these localities (Table 1). Species located in Boyacá (Fig. 3a) showed high quantities of the flavonoids 3-methoxy quercetin and hesperetin (Table 1), whereas species from Cundinamarca (Fig. 3a) were characterized by high quantities of dimeric flavonoids such as 8,8’-methylene-bisquercetin and a putative quercetin trimer (Table 1). The grouping of species in the páramos of Central and Western Cordillera of Colombia (Fig. 3a) was the only one in which the discriminatory substances were mainly diterpenes of the *ent-*kaurane type, such as *ent-*kaurenoic acid and gradiflorolic acid (Table 1). Lastly, the species located in the páramos of Venezuela (Fig. 3a) were characterized by the presence of high quantities of glycosylated flavonoids, such as quercetin-3-*O*-galactoside, which represents the main discriminatory substance of this group (Table 1). The accumulation of this flavonoid in high quantities in most of the species from Venezuela and the high quantities of 3-methoxy quercetin in the species from Colombia (especially those located in Boyacá) were the main reasons why these two groups of species (Colombian and Venezuelan) form two well-separated clusters in the OPLS-DA (Fig. 3b).

**Table 1:** Main discriminatory substances of the geographical origin of species from the genus *Espeletia* analyzed by UHPLC-UV-HRMS.

VarID	VIP	[M - H]^−^	Rt (min)	Molecular formula	Substance or chemical class
**Venezuela**					
163	7.60	463.08821	8.05	C_21_H_20_O_12_	quercetin-3-*O*-galactoside^*^
221	7.44	609.12463	10.06	C_30_H_26_O_14_	quercetin-3-*O*-(3,4-dihydroxy-*Z*-cinnamoyl)-(2)-α-L-rhamnopyranoside
169	5.28	625.11994	9.30	C_30_H_26_O_15_	quercetin 3-*O*-(6′′-*O*-caffeoyl)-galactoside
13	4.75	515.11926	6.32	C_25_H_24_O_12_	di-caffeoylquinic acid isomer
3	4.25	301.03448	10.40	C_15_H_10_O_7_	quercetin^*^
9	4.21	271.06125	11.46	C_15_H_12_O_5_	pinobanksin
160	4.17	191.05501	0.51	C_7_H_12_O_6_	quinic acid^*^
168	2.99	635.16179	7.86	C_29_H_32_O_16_	n.i.
225	2.57	771.17785	9.28	C_36_H_36_O_19_	n.i.
181	2.37	433.07758	8.40	C_20_H_18_O_11_	quercetin-3-*O*-arabinoside
**Santander/Norte de Santander**					
115	5.81	617.05769	11.11	C_30_H_18_O_15_	n.i.
405	4.30	477.35855	25.30	C_29_H_50_O_5_	putative triterpene
846	3.27	317.02998	6.37	C_15_H_10_O_8_	n.i.
845	3.26	505.38971	27.25	C_31_H_54_O_5_	putative triterpene
38	2.98	327.21800	12.05	C_18_H_30_O_5_	n.i.
947	2.72	491.37317	26.30	C_30_H_52_O_5_	putative triterpene
496	2.33	917.08438	13.21	—	n.i.
101	1.12	719.21900	8.65	—	n.i.
**Boyacá**					
1	10.58	315.05038	10.95	C_16_H_12_O_7_	3-methoxy quercetin^*^
179	9.13	301.07114	13.02	C_16_H_14_O_6_	hesperetin
455	3.05	533.09363	7.90	C_24_H_22_O_14_	di-caffeoylaltraric acid isomer
177	2.66	787.17261	8.45	C_36_H_30_O_20_	n.i.
1296	2.09	695.12524	9.40	C_33_H_28_O_17_	tri-caffeoylaltraric acid isomer
617	1.39	463.08900	9.04	C_21_H_20_O_12_	putative glycosilated flavonoid
58	1.25	325.18400	18.87	C_21_H_26_O_3_	n.i.
759	1.23	711.14280	7.49	—	n.i.
**Cundinamarca**					
366	4.68	629.09403	11.29	—	putative flavonoid
33	4.49	329.23300	12.73	C_18_H_34_O_5_	putative flavonoid
6	4.13	615.07758	14.15	C_31_H_20_O_14_	8,8″-methylene-bisquercetin
23	3.56	915.10504	15.65	C_46_H_28_O_21_	putative quercetin trimer
170	2.66	295.22800	18.90	C_18_H_32_O_3_	n.i.
15	2.10	587.08316	13.70	C_30_H_20_O_13_	putative flavanone-flavonol dimer
433	1.63	929.12200	13.52	—	n.i.
118	1.55	293.21233	19.62	C_18_H_32_O_3_	n.i.
**Central and Western Cordillera**					
42	4.88	695.12512	9.94	C_33_H_28_O_17_	tri-caffeoylaltraric acid isomer
164	4.61	315.19577	15.51	C_20_H_28_O_3_	*ent-*12-hydroxy-kaura-9(11),16-dien-18-oic acid
232	4.41	313.18039	14.77	C_20_H_26_O_3_	*ent-*12-oxo-kaura-9(11),16-dien-18-oic acid
1240	4.38	335.22278	14.25	C_20_H_32_O_4_	putative diterpene
362	3.97	317.21155	18.16	C_20_H_30_O_3_	grandiflorolic acid
12	3.59	301.21680	22.90	C_20_H_30_O_2_	*ent-*kaurenoic acid^*^
491	3.17	315.19630	17.67	C_20_H_28_O_3_	putative diterpene
1855	3.11	603.26630	12.44	C_28_H_44_O_14_	n.i.
1879	2.46	387.25363	23.34	C_24_H_36_O_4_	*ent-*15-*O*-iso-butiroxy-kaur-16-en-19-oic acid
1642	2.41	399.25351	24.24	C_25_H_36_O_4_	*ent-*15-*O*-senecioxy-kaur-16-en-19-oic acid

*Reference compound used to confirm identification. n.i.: not identified.

### Phytochemical characterization of *Espeletia*

A comprehensive phytochemical characterization of 120 samples of *Espeletia* allowed us to propose the identities of 46 compounds (Table S1). These compounds were detected in most of the analyzed samples and were identified with varying levels of confidence according to the Metabolomics Standards Initiative (MSI)^24^. Fifteen compounds were unambiguously identified by comparing chromatographic retention times (R_t_), UV spectra, accurate mass measurements and MS^2^ fragmentation patterns with those of the available reference compounds, corresponding to the MSI level 1. The identity of 25 putatively annotated compounds, belonging to MSI level 2, could be proposed based on the comparison of accurate mass measurements, UV spectra, MS^2^ fragmentation patterns, chemotaxonomic information and online database searches. On the other hand, the chemical classes of six additional substances, belonging to MSI level 3, were tentatively proposed based on accurate mass comparisons, MS^2^ fragmentation patterns and/or UV spectra (Table S1). The identified compounds can be grouped into five major chemical classes: *trans*-cinnamic acid derivatives, flavonoids, diterpenes, triterpenes and sesquiterpene lactones (Table S1). The chemical structures (Fig. S1) and detailed mass spectrometry information used to identify compounds reported in Table S1 are presented in the Supplementary Information (SI).

## Discussion

As core finding, this study reports the correlation between the metabolome and the biogeographic history of the genus *Espeletia* ^5^. According to recently published studies based on past climate fluctuations^1, 2^ and species distribution patterns^5^, *Espeletia* originated from the western or southwestern side of the Venezuelan Cordillera de Mérida during the early Pleistocene^5^ (Fig. 1). Subsequently, long before the last glaciation in the early or middle Pleistocene period, one branch of the population migrated towards the southwest, reaching the Eastern Cordillera of the Colombian Andes and colonizing the páramos of Santander and Norte de Santander (Fig. 1). Considering that the Táchira depression (at the border of both countries) forms a deep valley of unsuitable conditions for any Espeletiinae, it is plausible that an initial population of *Espeletia* crossed this depression during the cold Pleistocene period when the upper forest line shifted from its current limit at 3200 m to 1800 m in altitude^1, 2^. Since then, with the rising temperatures after the Pleistocene glaciations, the Colombian species have been evolving in isolation from their Venezuelan congeners, therefore explaining the biogeographical limit between Colombian and Venezuelan populations as well as the metabolic and genetic^18^ differences between species from the two countries. From the páramos of Santander and Norte de Santander, the genus continued to diversify, migrating southwards to the páramos of Boyacá and Cundinamarca (Fig. 1). These three orographic nuclei (Santander/N. de Santander, Boyacá and Cundinamarca) (Fig. 1) are the three main centers of *Espeletia* speciation in the Eastern Cordillera of Colombia, which support the characteristic chemical fingerprints of *Espeletia* species from these three adjacent localities (Fig. 3b). The current phylogeny (based on ITS, ETS, rp116 and AFLP molecular markers) of the subtribe Espeletiinae also supported these three páramo massifs as the main centers of biogeographic diversification in the Colombian Eastern Cordillera^18^. The metabolic differences observed between species from the Central and Western Cordillera of Colombia and their counterparts in the Eastern Cordillera (Fig. 3b) can also be used to trace the biogeographic diversification of *Espeletia*. According to the literature^5^, it was only during one of the last glaciations, certainly during the wet late glacial 14,000/12,000 BP–10,000 BP that *Espeletia* was able to spread to the Central Cordillera, where one branch migrated to northern Ecuador and another colonized the páramos of the Western Cordillera of Colombia^5, 25^ (Fig. 1). With the timberline rising considerably during the Holocene, páramos and their populations of *Espeletia* were reduced to a few isolated mountain systems. Species located in different páramo massifs lost contact with their counterparts in other páramos, preventing a continuous genetic flow^5^ and suggesting that *Espeletia* lineages modified their metabolic capacities as they independently evolved after the Pleistocene glaciations. This is further supported by the limitations of long-distance pollination and seed dispersal that the genus *Espeletia* faces^26–28^, which may be the main underlying cause of species grouping according to their biogeography, as shown here on the metabolic level and as previously reported on the genetic level at a higher geographical scale^18^. In some cases, two species located in distant páramos may display similar metabolic fingerprints. The two Venezuelan taxa, *E. schultzii* × *E. jajoensis* (100) and *E. ulotricha* (117) (Fig. 3a) were grouped with the Cundinamarca cluster most likely due to chemical convergence resulting from similar environmental conditions or selective forces acting on respective localities. However, additional analyses are necessary to shed light on this issue.

Interestingly, the current phylogeny of the subtribe Espeletiinae based on ITS, ETS, rpl16 and AFLPs markers^18^ also showed a strong geographic structure with two main clades, one clade of primarily Venezuelan species that included a few neighboring Colombian species (mainly from the genus *Tamania* and *Libanothamnus*) and a second clade including most of the Colombian species^18^. Therefore, based on molecular markers, only a country-level discrimination of the samples was achieved. In the current metabolomics approach, in addition to the country-level discrimination, we found a finer discrimination of the samples related to the species diversification centers. As stated by Diazgranados and Barber (2017), a pattern of phylogenetic clustering occurred within the Colombian clade, where species from the same páramo massif (sympatric species) tend to be closely related^18^. Our results support this finding as similar metabolic fingerprints also categorized species from the same massif, suggesting that species from the genus *Espeletia* started to accumulate certain metabolites (Table 1) in higher amounts as they respectively colonized the five major Colombian páramo massifs.

The phylogenetic clustering of species according to their biogeography is not restricted to the genus *Espeletia* or the subtribe Espeletiinae. The phytogeographical classification of Colombian páramos based on the distribution of angiosperm flora revealed that Colombian páramos can be divided into five major biogeographical units similar to the diversification centers of the genus *Espeletia* considered here, where characteristic angiosperm vegetation occurs^10^. Therefore, we believe that the correlation between the metabolome of *Espeletia* and its biogeography can analogously be applied to other plant groups of the páramo ecosystem and to plants belonging to other biogeographically related ecosystems (e.g., oceanic islands)^3^. This approach may be especially relevant in groups that also underwent rapid adaptive radiations such as the silversword alliance (Asteraceae, Madiinae) in Hawaii. Previous studies based on classical phytochemical approaches reported a highly similar flavonoid profile among mainland taxa and their counterparts in Hawaii with varying levels of *O*-glycosylation and *O*-methylation^29^. Similarly, a geographic variation of flavonoids from *Arnica cordifolia* Hook. (Asteraceae) was reported to occur between northern and southern populations in the eastern United States, suggesting the occurrence of two major geographically isolated populations during the Pleistocene glaciations^30^. In the previous study, the presence of glycosylated flavonoids was suggested to be an ancestral character relative to the presence of methoxylated flavonoids, which mirror our results, as glycosylated flavonoids were the main discriminatory substances (Table 1) of the ancestral species from Venezuela^5, 18^.

Despite the clear quantitative metabolic differences of *Espeletia* species between localities, this genus has a rather homogeneous qualitative chemical composition. Most of the compounds identified in the present study (Table S1) were detected in all plant samples at different concentrations. This could be a consequence of the short diversification times of the genus *Espeletia* ^2, 16, 25^ and of the fact that all Espeletiinae species are derived from a single ancestor^18^, thus explaining the need for supervised statistical methods to identify these differences.

Of the 46 identified compounds, 19 constitute new reports for the subtribe Espeletiinae and 21 for the genus *Espeletia* (Table S1), which indicates that the secondary metabolite chemistry of this group of plants is still largely unexplored. Some of the reported metabolites show high chemosystematic significance, including the sesquiterpene lactones longipilin acetate and polymatin B^31^ and the three caffeoylaltraric acid isomers identified^32^ (Table S1). This last class of compounds has been previously reported only in the genus *Smallanthus* Mack^33^, which along with the genus *Ichtyothere* Mart., corresponded to the sister groups of the subtribe Espeletiinae based on their molecular markers^34^. Interestingly, *ent-*kaurane-type diterpenes (Table S1) were identified in all plant samples, confirming previous chemotaxonomic studies that hypothesized that all species of the genus *Espeletia* biosynthesize such compounds^35^.

In this study, the UHPLC-UV-HRMS-based metabolic fingerprints of nine hybrids and their parent species were also assessed. However, no robust conclusion can be drawn concerning their chemotaxonomic relatedness as most of the analyzed hybrids were collected in different locations and at different times than their parent species, which hindered an accurate comparison as we cannot rule out the effect of environmental factors on species-chemical composition. However, because the objective of this study was not chemotaxonomical classification but rather to provide metabolomic evidence for biogeographic isolation of *Espeletia*, an alternative strategy was employed when collecting plant samples (see methods) to overcome the environmental effects on the correlation between metabolomics and biogeography. Furthermore, the NMDS analysis confirmed that among the different environmental factors considered (locality, season, elevation, month and year of collection), only the locality and year of collection showed a statistically significant influence on the species’ metabolic fingerprints, while the season, elevation and month of collection had no significant influence.

Although the year of collection appeared to be statistically significant, this result must be interpreted with caution, considering that in certain years we could only collect samples from one locality due to logistic challenges. In 2010, we predominantly collected Venezuelan samples, while in 2007 we only collected samples from Boyacá and Cundinamarca. The irregularities in the collection schedule unavoidably added bias to its correlation with the metabolomics profile because the locality, the most important factor, was made implicit during those respective years. An additional NMDS analysis (Fig. S2) considering three páramo massifs (Venezuela, Santander/North of Santander and Central/Western Cordillera), where all samples were collected in the same year, pointed to locality as the only factor with a significant influence (*p* = 0.0027) on the species’ metabolic fingerprints, while the specific year had no significant influence (*p* = 0.4). These data supported the results obtained by OPLS-DA.

In conclusion, the current study offers the first metabolomic report to reveal subtle biogeographical trends in plant diversification. This study demonstrates that *Espeletia* lineages can be distinguished by means of different metabolic fingerprints and that each lineage possesses a characteristic fingerprint correlating to the country of origin on a global scale and to the páramo massif on a regional scale. We identified distinctive patterns in the accumulation of secondary metabolites according to the main diversification centers of *Espeletia*. In addition, a comprehensive phytochemical characterization of 52 species, 72 individual taxa and 120 plant samples including biological replicates was carried out to putatively identify 46 compounds, 19 of them previously unreported in the subtribe Espeletiinae and 21 in the genus *Espeletia*. We included 73% of the reported species for this genus, and out of the 72 individual taxa analyzed, 60 were studied for the first time (83%). These results suggest that *Espeletia* lineages modified their metabolic capacities as they evolved independently after the Pleistocene glaciations and that secondary metabolites may have played an important role in the diversification and adaptive success of *Espeletia* to the Andean páramos, offering a phytochemical perspective on an Andean adaptive radiation.

## Methods

### Plant material

Young leaves of 52 known species, nine hybrids, 11 new species in the process of taxonomic description and 48 biological replicates (samples obtained from different individuals from the same species) of *Espeletia* (for a total of 120 plant samples, Table S2) were collected from the páramos of Colombia and Venezuela between December 2007 and August 2011. All collected plants were identified by Dr. Mauricio Diazgranados (Royal Botanic Gardens Kew, Ardingly, West Sussex, UK). Information about the exact location and elevation of the collection sites, the number of biological replicates and the season of collection for each species are reported in Table S2. All collected plant samples were immediately deposited inside resealable zipper storage bags containing silica gel beads with a humidity indicator. The same samples used in this study were also used in molecular studies to reconstruct the phylogeny of the group^18^. Contaminated samples were excluded from all the analyses. The collected species represent 73% of the recognized species in *Espeletia* and covered the entire range of geographic distribution of this genus. Vouchers of the Colombian species were deposited at the COL and ANDES herbaria, and the Venezuelan species were deposited at the MER herbarium according to the collection numbers reported in Table S2.

Considering that samples could not be collected at the same time and day due to the large geographical distance and difficulty in accessing the páramos of interest, we used an alternative approach to minimize the environmental effects on the metabolome of the analyzed samples and to accurately assess the significance of the locality of collection in the species’ metabolic fingerprints. Plants were collected in different months over four years (between 2007 and 2011, Table S2), and for each geographical locality, different individuals were collected on different dates. Subsequently, through NMDS and OPLS-DA (see below), the metabolome of all samples was used along with the collection date and elevation to find possible correlations, the results of which were negative (Figs 4 and S3).

To investigate any potential relationship between the seasonality and the metabolic fingerprint of species, we linked the collection sites with the climatological information of the closest páramos with available climate data. We then assigned the most likely season to the collection date based on Rangel (2000). Seasons were coded as “Dry season”, “Rainy season”, “Rainy season to Dry season” and “Dry season to Rainy season”^8^. We ran an NMDS with the resulting table but no correlation was found (Fig. 4). An OPLS-DA considering the season of collection as the Y variable was also performed (Fig. S4) and confirmed the NMDS results. There was no association between the season and metabolic fingerprint.

### Sample preparation

The extraction procedure used in this study was based on the detailed protocol for large-scale untargeted metabolomics of plant tissues reported by De Vos *et al*.^36^ with some modifications. Fifteen milligrams of leaf tissue was ground to a fine powder in liquid nitrogen using a mortar and pestle and was then extracted with 1.5 mL of HPLC-grade EtOH-H_2_O (7:3, v/v) solution in an ultrasonic bath for 15 min at 25 °C using a frequency of 40 kHz. After extraction, the samples were centrifuged at 19,975 × *g* for 10 min. The supernatant was partitioned with 0.5 mL of *n-*hexane, and the aqueous layer was filtered through a 0.2 µm PTFE membrane filter. All solvents were HPLC grade and a new membrane filter was used for each extract.

### UHPLC-UV-HRMS analysis

The UHPLC-UV-HRMS analyses were performed on an Accela UHPLC (Thermo Scientific, USA) apparatus with an 80 Hz photodiode array detector (PDA) coupled to a heated electrospray ionization source (HESI) and an Exactive Plus mass spectrometer with an Orbitrap analyzer (Thermo Scientific).

Each plant extract (10 µL) was injected and chromatographed on a C18 column (Hypersil Gold, 50 × 2.1 mm, Thermo Scientific) with a C18 Security Guard^TM^ Ultra cartridge (Phenomenex, USA). Solvent blanks were analyzed for comparison. The mobile phase consisted of purified water with 0.1% of formic acid (line A) and acetonitrile (line C). Separation was performed at a flow rate of 300 µL/min. The gradient started at 5% C and 95% A for the first 3 min and increased linearly to 100% C over 30 min. Use of 100% C continued for 5 min before the mobile phase composition returned to 5% C and 95% A over the following 5 min. The column temperature was maintained at 30 °C, and the tray temperature was kept at 10 °C. The PDA detector was set to record between 200 and 600 nm.

High-resolution mass spectrometry was carried out simultaneously in positive and negative ionization modes at a resolution of 70,000 full width at half maximum (FWHM). A capillary temperature of 300 °C was employed along with a spray voltage of +3.6 kV and −3.2 kV for each ionization mode. The mass range from 150 to 2,000 *m/z* was acquired. All plant samples were randomly analyzed and one extract was injected four times, one at the beginning, two in the middle and one at the end of the chromatographic sequence, to check for reproducibility (Fig. S5).

### Data preprocessing and biogeography correlation

Each set of chromatographic raw data were split into two sets according to the ionization mode (positive and negative) using the RecalOffline and Xcalibur (Thermo Scientific) software and subsequently exported to the software MZmine 2.10 (MZmine VTT, Finland), in which data preprocessing was performed. MZmine constitutes a framework for differential analysis of mass spectrometry data that allows several preprocessing steps, such as noise filtering, peak detection, deisotoping, alignment, identification and normalization, to be performed^37^.

To separate the compound signals from instrumental or chemical noise signals, a 1.0E6 noise threshold was employed using the exact mass algorithm. Noise peaks resulting from the Fourier transform function were removed using the Lorentzian function extended as HRMS shoulder peaks filter. For peak detection, the chromatogram builder function was used with a minimum time span set to 0.3 min, a minimum height of 1.5E6 and a 0.002 *m/z* tolerance. Chromatogram deconvolution was performed using the local minimum search algorithm (minimum relative height = 15%, minimum absolute height = 1.5E6, and minimum ratio of peak top/edge = 5), which finds the local minima in the chromatogram as border points between individual peaks^37^. Isotopes originating from the same compound were grouped using the isotopic peaks grouper function to avoid considering isotopes as different compounds^38^. All peak lists were aligned using the joint aligner method and the following parameters: *m*/*z* tolerance = 0.002 *m*/*z*, weight for *m*/*z* = 15, retention time tolerance = 0.2 absolute (min), weight for RT = 10 and a minimum score of 65%. Peaks not detected after alignment due to low intensity, poor quality or a mistake in peak detection were detected using the peak finder function with an intensity tolerance of 1%, a mass tolerance of 0.002 *m/z* and a retention time tolerance of 0.2 min. Adduct searching was performed according to each ionization mode. Finally, a secondary metabolite library comprising all chemical compounds reported in the subtribe Espeletiinae was created (see below). The data were inserted in MZmine and employed as a custom database, along with an in-house database (AsterDB, www.asterbiochem.org/asterdb) containing ca. 2,500 chemical structures reported in the Asteraceae family. Additional information regarding UHPLC-UV-HRMS and MZmine parameters can be found elsewere^20, 39^.

The data matrix obtained after MZmine preprocessing was transferred to a spreadsheet where the peaks detected in the blank were subtracted from each plant sample to remove interfering variables. This data matrix was scaled by the Pareto method, which keeps the data structure partially intact (major peaks in each sample remain) while reducing the relative importance of large values^40^. After scaling, the data matrix was submitted to unsupervised and supervised multivariate methods. Unsupervised methods were performed in the software R 3.0.3 (R Foundation for Statistical Computing, Austria). Non-metric multidimensional scaling was performed in the R package *vegan* using the Bray-Curtis dissimilarity index. Heatmaps were produced using the package *gplots*, considering only the top ranked OPLS-DA mass features (identified based on the criteria described below), while HCA with multiscale bootstrap resampling was performed with the package *pvclust* ^41^. We employed Ward’s minimum variance method as the clustering algorithm with a Euclidean distance. To assess the reliability of the clusters, 10,000 multiscale bootstrap replicates were performed considering only approximately unbiased *p*-values (AU), as they provide more accurate results than traditional bootstrapping by correcting the bias caused by a constant sample size^41^.

After performing the previous exploratory data analysis, a supervised analysis by OPLS-DA was carried out (SIMCA P 13.0.3.0, Umetrics AB, Sweden) to establish correlations between species metabolic fingerprints and their centers of biogeographic diversification. The OPLS-DA method separates the systematic variation in *X* (chromatographic peaks) into correlated and uncorrelated variables to *Y* (biogeographic centers)^42^. We considered three different criteria to identify the discriminating variables: (1) a variable importance in the projection value greater than 1.0, (2) a significant contribution in the loadings plot, and (3) a high magnitude and reliability jack-knifed confidence intervals in the loading or coefficient plots. Prior to the OPLS-DA analysis, the data matrix was scaled by the Pareto method. All multivariate analyses were performed with datasets generated in positive and negative ionization modes and with a combined dataset including both ionization modes. For each dataset (positive, negative or combined), additional analyses were performed by transforming all chromatographic peak areas into binary variables to explore correlations based only on the presence or absence of metabolites. In all cases, similar results were obtained (available upon request), but because the negative mode dataset displayed higher R^2^ and Q^2^ values and because most of the discriminating substances preferably ionized in such mode, this mode was chosen for further analysis. The performance of the OPLS-DA model was assessed using two different methods. The first method consisted of randomly splitting the data in two groups. Sixty-seven percent of the observations (82 samples) were used for building an additional model and the remaining 33% (37 samples) for external validation. A similar Q^2^ value to the former model was achieved and an adequate percentage of correctly classified instances were obtained. In the second method, we built ten additional models with different raw permutations as described by Triba *et al*.^43^ to check the stability of the Q^2^ value, which also validated our results^43^.

### Dereplication of plant extracts

Compounds were tentatively identified based on a comparison of the accurate mass measurements, UV spectra, MS^2^ fragmentation patterns, chemotaxonomy and online database searches. Accurate mass comparisons were performed relative to the theoretical monoisotopic mass (<3 ppm accuracy) of the secondary metabolites reported in the subtribe Espeletiinae, in the family Asteraceae, and those from the Dictionary of Natural Products (DNP, http://dnp.chemnetbase.com). We built a database containing all secondary metabolite reports of species from the subtribe Espeletiinae as follows: data collection using SciFinder (Chemical Abstract Service, USA), chemical structure drawing using MarvinSketch 14.9.15.0 (ChemAxon Ltd., Hungary) and database creation, handling and visualization using JChem for Excel (ChemAxon Ltd., Hungary). The information of this database (AsterDB) is freely available at www.asterbiochem.org/asterdb. To perform dereplication, this database was coupled to MZmine.

To confirm the peak assignments we performed MS^2^ experiments in a UHPLC-UV-HRMS HCD (higher-energy collisional dissociation) cell with a normalized collision energy of 35 units and a resolution of 35,000 FWHM. Additionally, the following 15 reference compounds were used to confirm identifications by comparing the retention times, accurate mass values, UV spectra and fragmentation patterns: quinic acid, 3-*O*-(*E*)-caffeoylquinic acid, protocatechuic acid, *p*-coumaric acid, rutin, quercetin-3-*O*-galactoside, 3,4-di-*O*-(*E)*-caffeoylquinic acid, 3,5-di-*O*-(*E)*-caffeoylquinic acid, 4,5-di-*O*-(*E)*-caffeoylquinic acid, quercitrin, quercetin, 3-methoxy quercetin, kaempferol, longipilin acetate and *ent-*kaurenoic acid. The presence of all dereplicated compounds in each species was reported considering a normalized intensity threshold value of 1.0E5, as experimental data suggest that lower intensity values may correspond to experimental noise and a deviation < 3 ppm between the experimental and theoretical monoisotopic mass of each compound.

### Data availability

The data that support the findings of this study are available from the MetaboLights repository (Study identifier: MTBLS445).

## Electronic supplementary material

Supplementary Information

Table S1.

Table S2.
