# Supplementary material for: Biogeography shaped the metabolome of the genus Espeletia: a phytochemical perspective on an Andean adaptive radiation
Source: Sci Rep. 2017 Aug 18;7:8835. doi: 10.1038/s41598-017-09431-7 (PMC5562832; doi:10.1038/s41598-017-09431-7)
Supplement: Supplementary file 1 — Supplementary Information [file 41598_2017_9431_MOESM1_ESM.pdf]

Biogeography shaped the metabolome of the genus *Espeletia*: a phytochemical perspective of on Andean adaptive radiation

Guillermo Federico Padilla-González[1], Mauricio Diazgranados[2], Fernando Batista Da Costa[1]*.

[1] AsterBioChem, Laboratory of Pharmacognosy, School of Pharmaceutical Sciences of Ribeirão Preto, University of São Paulo, Ribeirão Preto, SP, Brazil.

[2] Natural Capital and Plant Health Department, Millennium Seed Bank, Royal Botanic Gardens Kew, Ardingly, West Sussex, RH17 6TN, UK

***** Corresponding author

E-mail: febcosta@fcfrp.usp.br (FBC)

Tel.: +55 (16) 3602 0661

Supplementary Information

Compounds annotations:

Trans-cinnamic acid derivatives

Eleven *trans*-cinnamic acid derivatives (excluding flavonoids) were identified based on comparisons with reference substances and according to spectral characteristics described in the literature[1,2]. Compound 3 (Table S1), corresponding to 3-*O*-(*E*)-caffeoylquinic acid (Fig. S1), presented a precursor ion at 353.08716 *m/z* in the negative mode of ionization and a product ion at 191.05502 *m/z*, suggesting a possible esterification of a caffeic acid unit with a quinic acid molecule. Its unambiguous identification was possible based on Rt and HRMS comparisons with a reference substance. Compounds 1, 2 and 4 (Table S1, Fig. S1), identified as quinic acid, protocatechuic acid and *p*-coumaric acid, respectively, were also identified based on Rt and spectral comparisons with reference substances. Four di-caffeoylquinic acid isomers (compounds 5, 13, 15 and 17, Table S1, Fig. S1) were detected in most of the species. Among them, compounds 13, 15 and 17 (Table S1, Fig. S1) were identified as 3,4-di-*O*-(*E)*-caffeoylquinic acid, 3,5-di-*O*-(*E)*-caffeoylquinic acid and 4,5-di-*O*-(*E)*-caffeoylquinic acid, respectively, based on comparisons with reference substances. Compound 5 (Table S1) was proposed as another di-

caffeoylquinic acid isomer, considering that such compound showed a precursor ion at 515.11926 *m/z* in negative mode with fragments at 353.08780, 191.05530 and 179.03407 *m/z*, which is in accordance with the literature[3]. Additionally, its UV spectrum presented two maximum absorptions in *ca.* 300 and 325 nm, which characterize chlorogenic acids. Compound 12 (Table S1, Fig. S1) was proposed as methyl 3-*O*-caffeoyl-4-*O*-feruloylquinate[4] based on its mass and UV spectra. This compound showed a precursor ion at 543.15009 *m/z* in the negative ionization mode and a UV spectrum characteristic of chlorogenic acids. In the positive mode of ionization this molecule presented extensive fragmentation with a parent ion at 545.16455 *m/z*. The fragment observed at 527.15363 *m/z* indicates a possible loss of a neutral molecule of H_2O, while the fragment at 351.10672 *m/z* suggests the loss of a water molecule and a feruloyl unit. Additionally, we observed two peaks at 177.05429 *m/z* and 163.03853 *m/z* originated after the loss of a caffeoylquinate unit in the first case and the loss of a feruloylquinate unit in the second.

Compounds 9, 19 and 20 (Table S1, Fig. S1) were identified as caffeoylaltraric acids based on their UV and mass spectra. Compound 9 was identified as a di-caffeoylaltraric acid isomer. This compound presented a precursor ion at 533.09363 *m/z* in the negative mode and fragments peaks at 371.06198 and 209.02956 *m/z*, corresponding to successive losses of one and two caffeoyl units, respectively (Table S1). According to literature reports a product ion at 209 *m/z* is characteristic of galactaric acid[5]. On the other hand, compounds 19 and 20 (Table S1, Fig. S1) were identified as two possible tri-caffeoylaltraric acid isomers[6]. These compounds presented an MS^2 spectrum characterized by three successive losses of caffeoyl units and a characteristic UV spectrum with two maxima at 300 and *ca.* 330 nm. Compounds 19 and 20 showed a parent ion at 695.12524 *m/z* and fragment ions at 533.09363, 371.06171 and 209.02954 *m/z* in the negative mode of ionization.

Flavonoids

Flavonoids correspond to the main class of secondary metabolites identified in *Espeletia*. These metabolites were identified in all plant samples and can be divided into three main chemical subclasses: 1) aglycones, 2) glycosylated and 3) esterified with caffeoyl or cinnamoyl units. Among the aglycones, compounds 22, 23 and 25 (Table S1, Fig. S1) were identified as quercetin, 3-methoxy quercetin and kaempferol, respectively, based on their spectral and Rt comparison with reference standards. Compound 24 (Table S1, Fig. S1), also an aglycone, was proposed as pinobanksin[7]. This compound presented a UV spectrum with two maximum at *ca.* 230 and 290 nm, which is in accordance with the UV absorption

values reported in the literature for the same compound[8]. Its MS^2 spectrum presented a deprotonated molecule at 271.06125 *m/z* and product ions at 165.01825, 151.00252, 119.04893 and 107.01253 *m/z*, which is in accordance with the MS^2 spectrum reported for pinobanksin[7]. Compound 26 (Table S1, Fig. S1) was identified as hesperetin based on accurate mass comparisons with data reported in databases (DNP and AsterDB) and spectral comparisons with literature data[9]. This compound presented two maxima UV abortions at 228 and 288 nm and fragment ions at 165.01829 *m/z* and 135.04392 *m/z*.

Compound 28 (Table S1, Fig. S1) was identified as 8,8''-methylene-bisquercetin considering that such compound presented a precursor ion at 615.07758 *m/z* and a fragment ion at 299.01920 *m/z* in the negative mode, corresponding to the loss of a quercetin unit and a methyl group. In the positive mode of ionization this compound presented a MS^2 spectrum with a precursor ion at 617.09143 *m/z* and several product ions at 327.04907, 317.06363, 316.05643, 315.04926, 303.04886bp, 302.04156, 301.03375, 287.05447 *m/z*, which is in accordance with literature reports[2]. Compounds 27 and 33 (Table S1) were putatively identified as a flavanone-flavonol dimer and a quercetin trimer, respectively, based on their mass spectra. Compound 27 presented a deprotonated molecule at 587.08316 *m/z* and a fragment ion at 299.01912 *m/z* putatively formed after the loss of a flavanone unit. Its UV spectra showed abortions at 232, 291, 360 (shoulder) nm. Compound 33 showed a parent ion at 915.10504 *m/z* in the negative mode of ionization and a product ion at 299.01923 *m/z*. Both compounds (27 and 33, Table S1) displayed the same product ion at 299 *m/z* exhibited by 8,8''-methylene-bisquercetin (compound 28, Table S1).

Among the glycosylated flavonoids, compounds 8, 10 and 16 (Table S1, Fig. S1) were unambiguously identified as rutin, quercetin-3-*O*-galactoside and quercitrin, respectively, based on the comparison of their retention time, accurate mass, UV and MS^2 spectra with those of the reference substances. This type of compounds is characterized by the presence of MS^2 spectra with neutral losses of the glycosyl unit, followed by an intense peak representing the flavonoid aglycone. Additionally, such compounds present UV spectra characteristic of flavonoids with maxima at *ca.* 255 and 350 nm. Compound 6 (Table S1, Fig. S1) was identified as quercetin-3,4'-di-*O*-glucoside[10], considering that such compound presented a precursor ion at 625.14148 *m/z* (negative mode) and two fragment ions with *m/z* values of 463.08838 and 301.03510, corresponding to the successive losses of two glycosyl units. Compound 7 (Table S1, Fig. S1) was proposed as quercetin 3-*O*-(6-malonyl-glucoside) 7-*O*-glucoside[11]. This compound presented a precursor ion at 711.14093 *m/z*, a product ion at 463.08780 *m/z*, corresponding probably to the loss of a manoylglucosyl unit and a base peak at 301.03522 *m/z*

corresponding to the aglycone. The same pattern of glycosyl losses and a subsequent peak representing the flavonoid aglycone was also observed in the MS^2 spectra of compounds 11 and 14 (Table S1, Fig. S1), identified as quercetin-3-*O*-arabinoside[12], isorhamnetin 3-glucoside[13], respectively.

Compounds 18 and 21 (Table S1, Fig. S1) correspond putatively to two flavonoids esterified with caffeoyl or cinnamoyl units based on their characteristic UV spectra, which presented the usual signals of chlorogenic acids (two maximum absorptions in *ca.* 300 and 325 nm), and fragmentation patterns. Compound 18 (Table S1, Fig. S1) was identified as quercetin 3-*O*-(6′′-*O*-caffeoyl)-glucopyranoside[14]. Such compound showed a protonated molecule at 627.13342 *m/z* and fragment ions at 303.04935 and 163.03860 *m/z* in the positive mode. Those fragment ions may correspond to the aglycone at 303 *m/z* and an ionized caffeoyl unit at 163 *m/z*. Compound 21 (Table S1, Fig. S1) was proposed as quercetin-3-*O*-(3,4-dihydroxy-*Z*-cinnamoyl)-(→2)-α-L-rhamnopyranoside, based on its accurate mass, UV and MS^2 spectra.

Diterpenes

The identity of eleven diterpenes was tentatively proposed based only on accurate mass comparisons with the previously reported metabolites in the genus *Espeletia* and in the subtribe Espeletiinae. Compound 41 (Table S1, Fig. S1) was unambiguously identified as *ent*-kaurenoic acid based on retention time and accurate mass comparisons with a reference substance. All the identified diterpenes (Compounds 29, 30, 32, 36-40, 42 and 43, Table S1, Fig. S1) correspond to *ent*-kaurane derivatives, which constitute the most commonly reported secondary metabolites in the subtribe Espeletiinae[15–17].

Triterpenes

The chemical class of three putative triterpenes (Compounds 44, 45 and 46, Table S1, Fig. S1) was tentatively proposed based on their accurate mass measurements and data base screening. It is worth mentioning that the identity of each of these compounds was not reported here, as additional information regarding the MS^2 fragmentation patterns of the several possible hits (all of them triterpenes) identified in the DNP lacks in the literature. These three compounds showed the same fragmentation pattern in the MS^2 experiments with an initial loss of 150.0683 Da to form the base peak and a subsequent peak at 149.05972 *m/z*. Additionally, as previous phytochemical studies with species of the genus *Espeletia* have

reported this class of metabolites it may be safe to assume its presence in some of the plant samples analyzed[18–20].

Sesquiterpene lactones

We identified three sesquiterpene lactones, namely fluctuadin, longipilin acetate, and polymatin B (Compounds 31, 34 and 35, Table S1, Fig. S1, respectively) based on accurate mass comparisons with a reference standard (compound 34, Table S1, Fig. S1) and with compounds previously reported in Espeletiinae and in the genus *Smallanthus* (compounds 31 and 35, Table S1, Fig. S1). Compounds 34 and 35 were previously isolated from the leaves of *E. killipii* and *E. tunjana* and show chemotaxonomical significance[20]. On the other hand, compound 31 has not been previously reported in the subtribe Espeletiinae, but it is commonly found in the genus *Smallanthus*[21], the sister group of Espeletiinae based on molecular markers[22].

References

1. Gobbo-Neto, L. & Lopes, N. P. Online identification of chlorogenic acids, sesquiterpene lactones, and flavonoids in the Brazilian arnica *Lychnophora ericoides* Mart. (Asteraceae) leaves by HPLC-DAD-MS and HPLC-DAD-MS/MS and a validated HPLC-DAD method for their simultaneous analysis. *J. Agric. Food Chem.* **56,** 1193–204 (2008).

2. Martucci, M. E. P., De Vos, R. C. H., Carollo, C. A. & Gobbo-Neto, L. Metabolomics as a potential chemotaxonomical tool: application in the genus *Vernonia* schreb. *PLoS One* **9,** e93149 (2014).

3. Clifford, M. N., Knight, S. & Kuhnert, N. Discriminating between the six isomers of dicaffeoylquinic acid by LC-MS^n. *J. Agric. Food Chem.* **53,** 3821–3832 (2005).

4. Jaiswal, R. & Kuhnert, N. How to identify and discriminate between the methyl quinates of chlorogenic acids by liquid chromatography-tandem mass spectrometry. *J. Mass Spectrom.* **46,** 269–281 (2011).

5. Ismail, I. S., Ito, H. & Yoshida, T. An ichthyotoxic procyanidin oligomer and four new hydroxy acid conjugates from the leaves of *Sandoricum koetjape*. *ACGC Chem. Res. Commun.* **21,** 13–19 (2007).

6. Takenaka, M. *et al.* Caffeic acid derivatives in the roots of yacon (*Smallanthus sonchifolius*). *J. Agric. Food Chem.* **51,** 793–796 (2003).

7. Kečkeš, S. *et al.* The determination of phenolic profiles of Serbian unifloral honeys using ultra-high-performance liquid chromatography/high resolution accurate mass spectrometry. *Food Chem.* **138,** 32–40 (2013).

8. Mao, W., Schuler, M. A. & Berenbaum, M. R. Honey constituents up-regulate detoxification and immunity genes in the western honey bee *Apis mellifera*. *Proc. Natl. Acad. Sci.* **110,** 8842–8846 (2013).

9. Tahri, W. *et al.* Phenolic profiling of the aerial part of *Chrysanthemum trifurcatum* using ultra high performance liquid chromatography coupled to Orbitrap high resolution mass spectrometry. *Anal. Methods* **8,** 3517–3527 (2016).

10. Jaramillo, K., Dawid, C., Hofmann, T., Fujimoto, Y. & Osorio, C. Identification of antioxidative flavonols and anthocyanins in *Sicana odorifera* fruit peel. *J. Agric. Food Chem.* **59,** 975–983 (2011).

11. Llorach, R., Martínez-Sánchez, A., Tomás-Barberán, F. A., Gil, M. I. & Ferreres, F. Characterisation of polyphenols and antioxidant properties of five lettuce varieties and escarole. *Food Chem.* **108,** 1028–1038 (2008).

12. Rainha, N. *et al.* HPLC–UV–ESI-MS analysis of phenolic compounds and antioxidant properties of *Hypericum undulatum* shoot cultures and wild-growing plants. *Phytochemistry* **86,** 83–91 (2013).

13. Mijowska, K., Ochmian, I. & Oszmiański, J. Impact of cluster zone leaf removal on grapes cv. regent polyphenol content by the UPLC-PDA/MS method. *Molecules* **21,** 1688 (2016).

14. Shigematsu, N., Kouno, I. & Kawano, N. Quercetin 3-(6’’-caffeoylgalactoside) from *Hydrocotyle sibthorpioides*. *Phytochemistry* **21,** 2156–2158 (1982).

15. Alvarenga, S. A. V., Ferreira, M. J. P., Rodrigues, G. V. & Emerenciano, V. P. A general survey and some taxonomic implications of diterpenes in the Asteraceae. *Bot. J. Linn. Soc.* **147,** 291–308 (2005).

16. Usubillaga, A., de Hernandez, J., Perez, N. & Kiriakidis, M. Kauranoid diterpenes in *Espletia* species. *Phytochemistry* **12,** 2999 (1973).

17. Bohlmann, F., Suding, H., Cuatrecasas, J., King, R. M. & Robinson, H. New diterpenes from subtribe Espeletiinae. *Phytochemistry* **19,** 267–271 (1980).

18. Tellez, A. N., Torrenegra, R. D., Pedrozo, J. & Gray, A. Cycloartan-2 beta-2-methyl butanoate isolated from genus *Espeletia* (Asteraceae). *Molecules* **3,** M49-U4 (1998).

19. Tellez, A. N., Torrenegra, R. D., Pedrozo, J. & Martínez, A. Cycloeucalen-3 beta-(2-methyl butanoate). New cycloeucalen isolated from the *Espeletia barclayana* Cuatr. (Asteraceae). *Molecules* **5,** M163-U6 (2000).

20. Torrenegra, R. D. & Tellez, A. N. Chemotaxonomic value of melampolides in *Espeletia* species (Asteraceae). *Biochem. Syst. Ecol.* **23,** 449–450 (1995).

21. Mercado, M. I., Coll Aráoz, M. V., Manrique, I., Grau, A. & Catalán, C. A. N. Variability in sesquiterpene lactones from the leaves of yacon (*Smallanthus sonchifolius*) accessions of different geographic origin. *Genet. Resour. Crop Evol.* **61,** 1209–1217 (2014).

22. Rauscher, J. T. Molecular phylogenetics of the *Espeletia* complex (Asteraceae): Evidence from nrDNA ITS sequences on the closest relatives of an andean adaptive radiation. *Am. J. Bot.* **89,** 1074–1084 (2002).

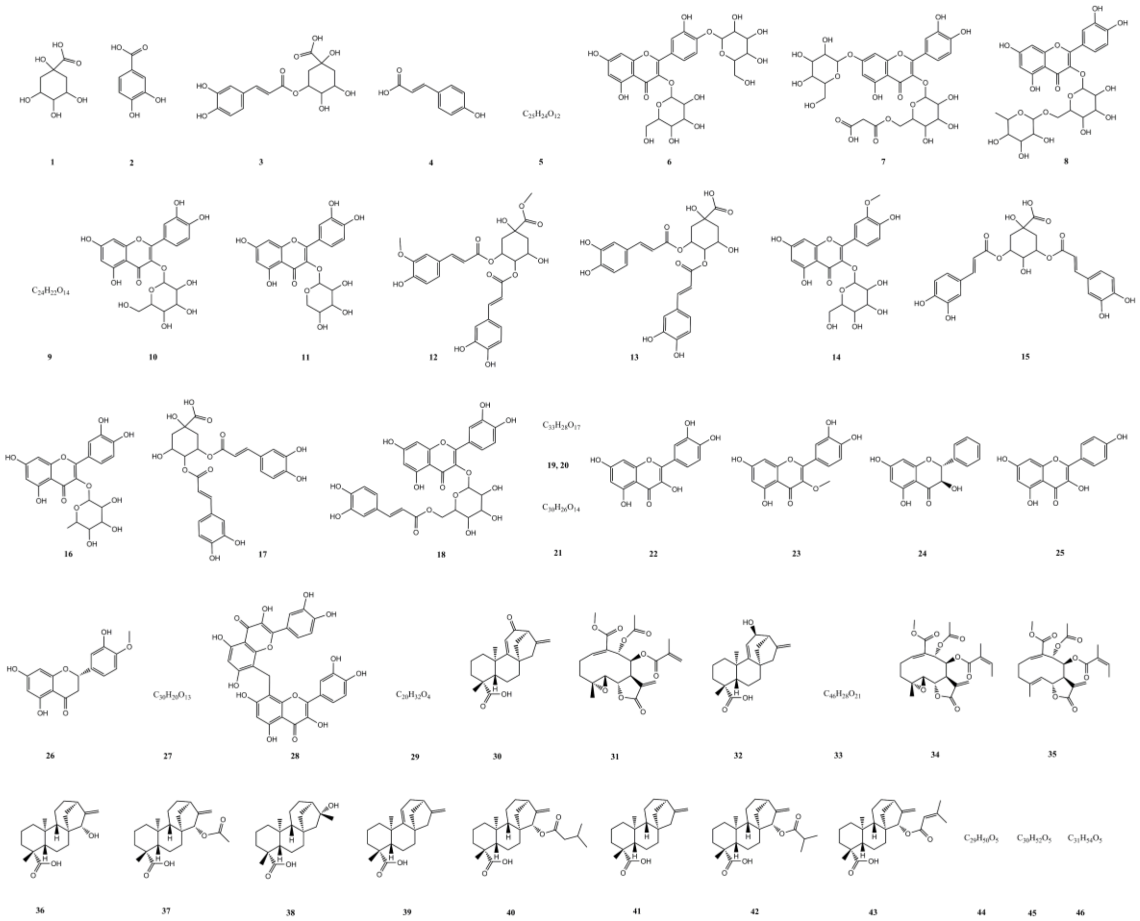

Fig. S1 Chemical structures or molecular formulae of identified substances in species of the genus *Espeletia* analyzed by UHPLC-UV-HRMS. See Table S1 for compound names.

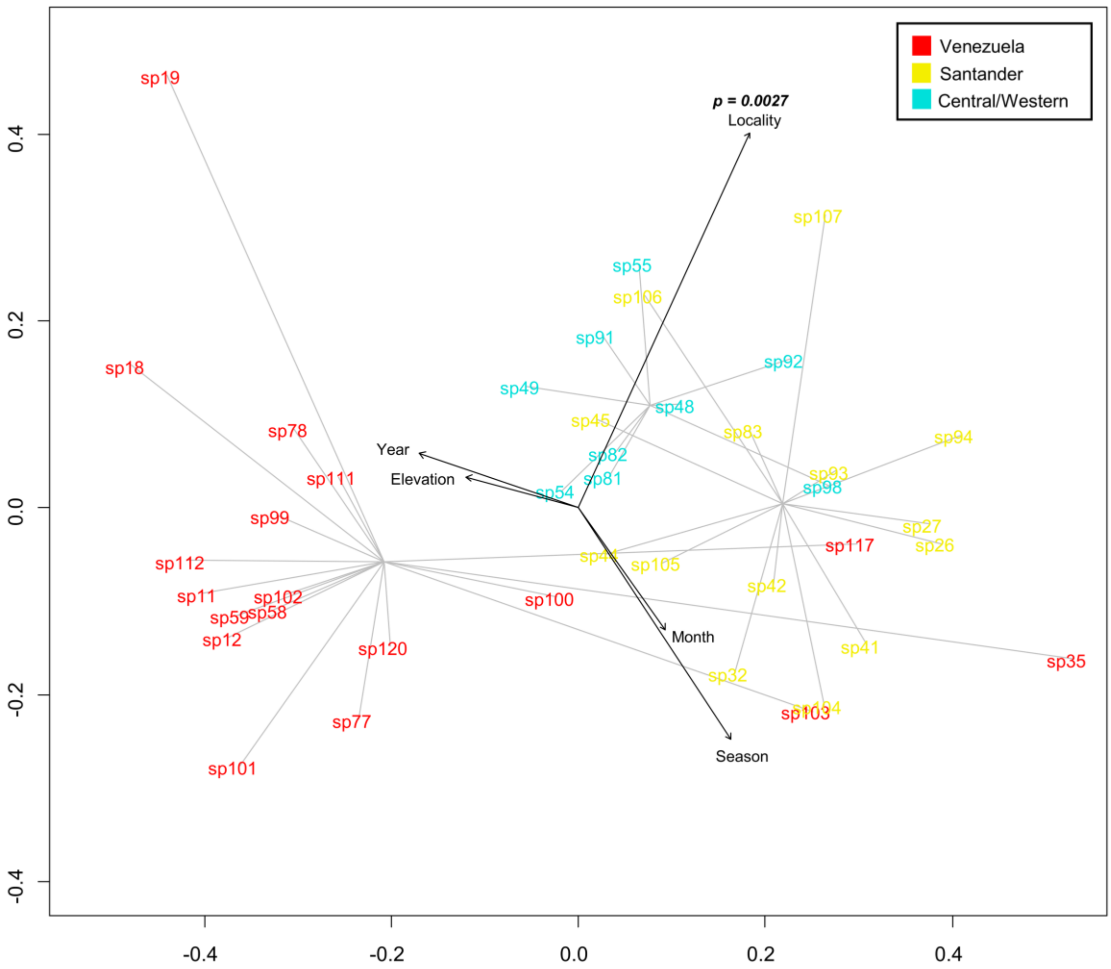

Fig. 2 NMDS based on metabolic fingerprinting in negative ionization mode of the species from the genus *Espeletia* collected in the páramos of Venezuela, Santander/N. de Santander and Central/W. Cordillera, showing the correlation of the environmental factors with the clustering of species by geographical origin.

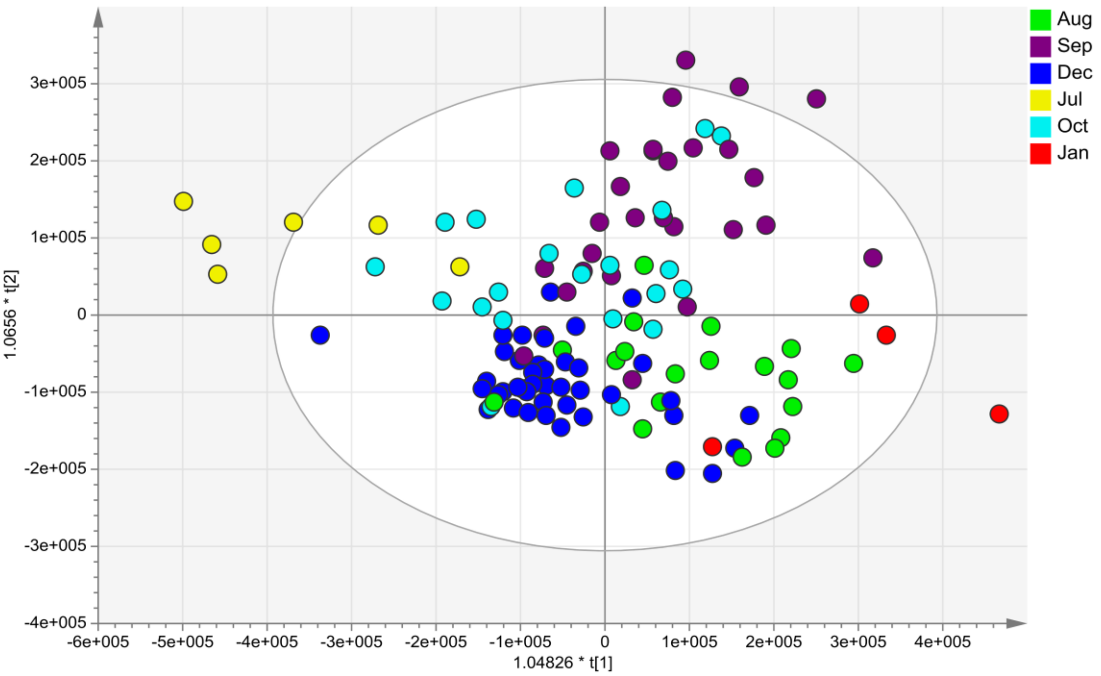

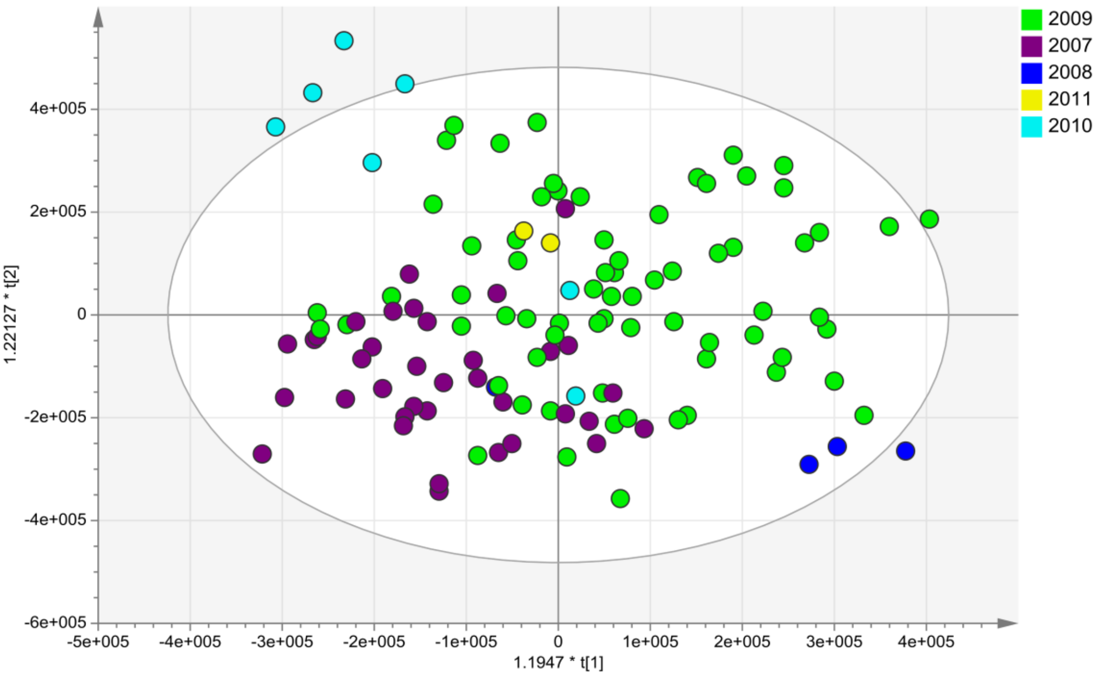

Fig. S3 OPLS-DA scores plot (upper panel: R^2 = 0.44, Q^2= 0.09; lower panel: R2 = 0.45, Q2= 0.22) of 120 plant samples of the genus *Espeletia* analyzed by UHPLC-UV-HRMS (negative mode), considering the month (upper panel) and year (lower panel) of collection as Y-variable. (Note: low R^2 and Q^2 values and poor separation indicate a null correlation).

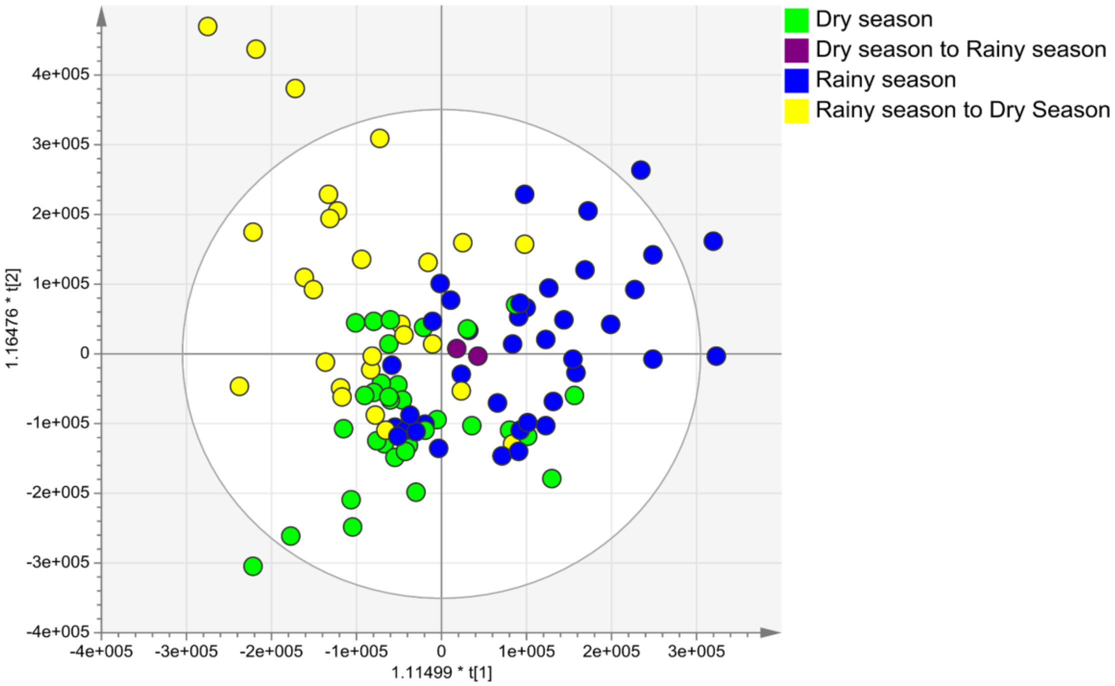

Fig. S4 OPLS-DA scores plot (R^2 = 0.26, Q^2= 0.05) of 120 plant samples of the genus *Espeletia* analyzed by UHPLC-UV-HRMS (negative mode), considering the season of collection as Y-variable. (Note: low R^2 and Q^2 values and poor separation indicate a null correlation).

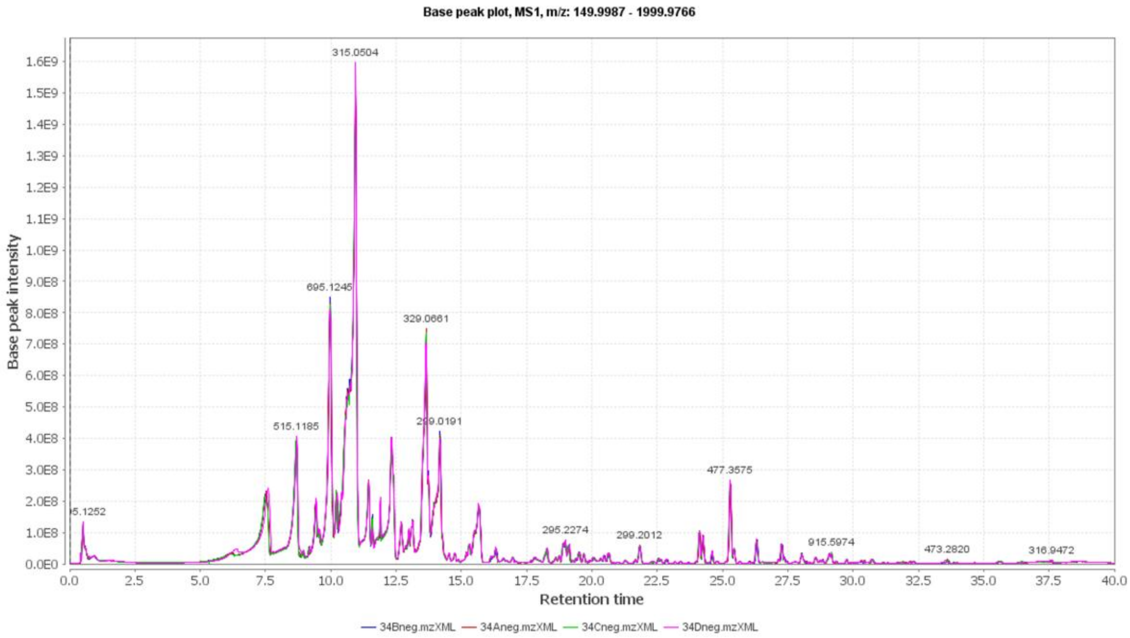

Fig. S5 Superimposed total ion current chromatogram in negative mode of four technical replicates of *Espeletia grandiflora* analyzed by UHPLC-UV-HRMS at different times along the chromatographic sequence.
